# A Canopy Information Measurement Method for Modern Standardized Apple Orchards Based on UAV Multimodal Information

**DOI:** 10.3390/s20102985

**Published:** 2020-05-25

**Authors:** Guoxiang Sun, Xiaochan Wang, Haihui Yang, Xianjie Zhang

**Affiliations:** 1 College of Engineering, Nanjing Agricultural University, Nanjing 210031, China; wangxiaochan@njau.edu.cn (X.W.); 2019212004@njau.edu.cn (H.Y.); 2018112036@njau.edu.cn (X.Z.); 2 Jiangsu Province Engineering Lab for Modern Intelligent Facilities of Agriculture Technology & Equipment, Nanjing 210031, China

**Keywords:** orchard, apple tree, canopy information, unmanned aerial vehicle (UAV), multispectral, multimodal

## Abstract

To make canopy information measurements in modern standardized apple orchards, a method for canopy information measurements based on unmanned aerial vehicle (UAV) multimodal information is proposed. Using a modern standardized apple orchard as the study object, a visual imaging system on a quadrotor UAV was used to collect canopy images in the apple orchard, and three-dimensional (3D) point-cloud models and vegetation index images of the orchard were generated with Pix4Dmapper software. A row and column detection method based on grayscale projection in orchard index images (RCGP) is proposed. Morphological information measurements of fruit tree canopies based on 3D point-cloud models are established, and a yield prediction model for fruit trees based on the UAV multimodal information is derived. The results are as follows: (1) When the ground sampling distance (GSD) was 2.13–6.69 cm/px, the accuracy of row detection in the orchard using the RCGP method was 100.00%. (2) With RCGP, the average accuracy of column detection based on grayscale images of the normalized green (NG) index was 98.71–100.00%. The hand-measured values of *H*, *S*_XOY_, and *V* of the fruit tree canopy were compared with those obtained with the UAV. The results showed that the coefficient of determination *R*^2^ was the most significant, which was 0.94, 0.94, and 0.91, respectively, and the relative average deviation (RAD_avg_) was minimal, which was 1.72%, 4.33%, and 7.90%, respectively, when the GSD was 2.13 cm/px. Yield prediction was modeled by the back-propagation artificial neural network prediction model using the color and textural characteristic values of fruit tree vegetation indices and the morphological characteristic values of point-cloud models. The R^2^ value between the predicted yield values and the measured values was 0.83–0.88, and the RAD value was 8.05–9.76%. These results show that the UAV-based canopy information measurement method in apple orchards proposed in this study can be applied to the remote evaluation of canopy 3D morphological information and can yield information about modern standardized orchards, thereby improving the level of orchard informatization. This method is thus valuable for the production management of modern standardized orchards.

## 1. Introduction

The canopy is the first part of the fruit tree exposed to light and the external environment and is the main place for photosynthesis and respiration. The shape, structure, volume, leaf area index, vegetation index, and other information of the fruit tree canopy not only reflect the growth status and yield potential of fruit trees [1] but also are important for decision-making about orchard canopy pruning [2,3], irrigation and fertilization [4], precision spraying [5], flowering management [6], and other key production steps [7].

Traditional fruit tree canopy information mainly relies on manual measurement methods based on previous experience. Based on field sampling data of orchard canopy information, survey results are extrapolated to the management scale and are used for reference [8]. Such traditional measurement and research methods have played an important role for quite a long time. With the development of intensive agriculture, traditional manual measurement methods have come to exhibit limitations in the rapid and accurate measurement of fruit tree canopy parameters. A low level of orchard informatization leads to problems such as low orchard production efficiency, low level of intelligent orchard management, and heavy pollution due to the large amounts of water, fertilizers, and pesticides/herbicides used. These shortcomings seriously restrict the development of precision production management in modern standardized orchards. It is urgently required to study canopy information measurement technologies in modern standardized orchards to lay a good foundation for the production management of modern standardized orchards.

With the development of sensor technology, the combination of unmanned aerial vehicles (UAVs) and visual imaging technology [9] allows the realization of nondestructive measurement of canopy information across large orchards, which can enable scientific and reliable decision-making for orchard production management. Hence, studying this technology will be key to the development of modern standardized orchards.

The main methods of measuring canopy information in large, modern standardized orchards are vehicle-mounted measurement systems [1,10] and UAV measurement systems [2,3,11]. A measurement system integrates ultrasound, a digital camera, a stereo camera, a time-of-flight (TOF) camera, a multispectral camera, a hyperspectral camera, two-dimensional (2D) light detection and ranging (LIDAR), three-dimensional (3D) LIDAR, high-resolution X-rays, and other technologies [7] to gather information about the orchard canopy 3D geometry, physiological indicators, and yield. Vehicle-mounted measurement systems have better measurement accuracy than UAV measurement systems, but they have lower measurement efficiency and a higher measurement cost than UAV measurement systems. Vehicle-mounted measurement systems are more suitable for the real-time orchard management practices, such as canopy target spraying [12], canopy pruning [2,3], and fruit positioning and picking [13], whereas the UAV measurement system is more suitable for information evaluation in orchard management, such as about canopy geometric morphology [14], biomass [15], diseases [16], water stress [17], vegetation indices [18], and yield [19,20].

At present, UAV visual imaging systems allow the establishment of multimodal information, such as orthophotographic maps, vegetation index images, digital surface models (DSMs), and 3D point-cloud models in large orchards, which have laid a good foundation for canopy information measurements in orchards. Scholars in this field have carried out a large body of research on two aspects: orchard canopy information extraction methods and orchard management. Studying UAV-based orchard canopy information extraction methods, Zarco-Tejada et al. [14] used orthorectification and DSMs of olive orchards to remotely evaluate the height of fruit trees. Koc-San et al. [11] used a UAV to collect citrus orchard images and proposed an automatic extraction method based on the circular Hough transform model for images and DSMs in citrus orchards. Comba et al. [21] used a UAV to collect 3D point-cloud models of orchards and proposed a method for 3D point-cloud model characteristic value extraction in hilly-terrain orchards. UAV measurement systems can also be used for orchard management, such as fruit tree canopy pruning, which can increase canopy light interception and canopy surface area and promote new growth of fruit trees, making fruits more readily pickable and possibly increasing the yield of the trees. To evaluate the effect of pruning on litchi orchards, Johansen et al. [3] used UAV to measure tree structure, such as canopy circumference, width, height, area, and plant projection coverage. Additionally, early diagnosis of orchard diseases and insect pests can improve the control effect of pests and diseases in orchards and can improve the quality and yield of fruits. To this end, through a UAV, Garcia-Ruiz et al. [16] collected multispectral canopy images in citrus orchards and established a diagnostic model of Huanglongbing (HLB) disease (citrus greening) in citrus orchards, which allowed them to classify fruit trees by HLB disease status. Through a UAV visual system, Park et al. [22] collected high-resolution thermal images of a peach orchard and established a diagnostic model for fruit tree water stress to guide the orchard irrigation management. As a UAV plant protection sprayer can improve the accuracy of target spraying and reduce the application amount, Cheng et al. [5] proposed an automatic segmentation method for canopy images in orchards and established an accurate spraying method based on canopy area. Meanwhile, UAV measurement technology has also been applied in a large body of research in field crops or plants, such as on cotton yield measurement [19], corn height measurement [23], barley biomass measurement [15], wheat height measurement [24], sorghum growth status measurement [25], sunflower yield measurement [26], field weed detection [27], and rapeseed yield measurement [20]. UAV measurement technology is highly efficient and nondestructive and is suitable for large-scale measurement of information about orchards and field crops. Compared with the ground measurement method [28], this technology is more suitable for applications in large, modern precision agricultural production management.

As modern standardized orchards must accommodate agricultural machinery and equipment, they need a large enough row spacing and plant spacing. A typical characteristic of modern standardized orchards is neat rows and columns, yet the presence of a few small trees or dead trees between fruit trees could affect the benefit of canopy segmentation. In this study, taking an apple orchard as the study object, a UAV visual imaging system was used to generate 3D point-cloud models and vegetation indices of the apple orchard. Automatic row and column detection methods were established based on vegetation index images, thereby achieving rapid segmentation. Based on the fruit tree point-cloud models, measurement methods for height, projection area, and projection volume, as well as yield prediction models, were established to achieve a remote evaluation of orchard canopy information. This paper provides a theoretical basis and technical reference for canopy information measurement in modern standardized orchards and will be helpful in orchard management.

## 2. Materials and Methods

### 2.1. UAV Measurement System for Canopy Information of Orchards

The UAV measurement system for modern standardized apple orchards mainly consisted of a Parrot Bluegrass UAV, Parrot Sequoia multispectral cameras, Pix4Dcapture flight control software, Pix4Dmapper postprocessing software, and a graphics workstation. The main technical parameters of the Parrot Bluegrass UAV were as follows: maximal take-off mass (MTOM) is 1.85 kg, battery life of 25 min, Parrot Skycontroller 2 with a control range of 2 km, and a vertical camera equipped with a built-in global positioning system (GPS), global navigation satellite system (GLONASS), altimeter, ultrasonic sensor, and optical flow vertical camera. The main technical parameters of the Parrot Sequoia multispectral camera are as follows: an RGB camera with the resolution of 16 megapixels (4608 × 3456 px); four global-shutter spectral-channel single-band cameras, including green (GRE, 550 ± 40 nm), red (RED, 660 ± 40 nm), red-edge (REG, 735 ± 10 nm), and near-infrared (NIR, 790 ± 40 nm), each with the resolution of 1.2 megapixels (1280 × 960 px) and the frame rate of 1 frame/s, it has four environmental light sensors, these sensors have the same band-pass filter as four single-band cameras, and pictures could be calibrated automatically according to the light conditions. The main functions of the Pix4Dcapture flight control software were as follows: UAV flight map selection, flight path planning, flight altitude, flight speed, horizontal coverage, vertical coverage, and other parameter settings, along with the display function of real-time status information parameters, such as the UAV battery, Parrot Skycontroller battery, memory, GPS, and operation processes. The main functions of the Pix4Dmapper postprocessing software were generating 3D point-cloud models, orthographic images, and DSM and vegetation index images based on the images collected by the UAV. The main technical parameters of the graphics workstation (HP Zhan 99 by Hewlett-Packard Corporation, Beijing, China) were as follows: a 6-core 12-thread Intel (R) Xeon (R) E-2176 M CPU @ 2.70 GHz, 32 GB of RAM, Intel (R) UHD Graphics P630 GPU and NVIDIA Quadro P600 4G graphics card. The operating system was Windows 10 Professional Workstation Edition, 64 bit. The software for calculating the 3D morphological information of the fruit tree canopy was Matlab 2017a (MathWorks Corporation, Natick, MA, USA).

The workflow of the UAV measurement system for canopy information in a modern standardized apple orchard is as follows: The first step was image acquisition. According to the selected orchard measurement area, Pix4Dcapture created a flight (including flight map boundary selection, flight path planning, flight altitude, flight speed, horizontal coverage, vertical coverage, and other parameter settings), the Parrot Bluegrass performed this flight, and the Parrot Sequoia multispectral camera collected multispectral images at each planned waypoint. The second step was the point-cloud output. Pix4Dmapper was used to read images collected by the flights, and according to the GPS information (longitude, latitude, altitude) of each waypoint, Pix4Dmapper carried out processing procedures, such as computing keypoints, computing matches, calibration, matching, and point-cloud densification, and finally output the PLY file of the point-cloud model of the orchard. The third step was the vegetation index output. According to GRE, RED, REG, and NIR orchard canopy orthophotos, Pix4Dmapper was used to generate various vegetation index images (such as normalized difference vegetation index (NDVI), normalized green (NG), green chlorophyll index (CIG), and renormalized DVI (RDVI)), and the orchard vegetation index images were output in JPG format (the type of vegetation index was set manually according to the actual situation). The fourth step was a row and column detection in the orchard canopy. The orchard point-cloud models and vegetation index images were read in Matlab, and the grayscale projection method based on 2D vegetation index images was used to realize the automatic row and column detection and segmentation of 2D/3D orchard canopy images. The point-cloud model of each fruit tree was saved in a 2D cell array. The fifth step was to calculate canopy information. According to the point-cloud model of each fruit tree, various morphological characteristic parameters were calculated, such as canopy height, canopy projection area, and circumscribed volume of the canopy. According to the vegetation index of each fruit tree, various color characteristic parameters were calculated, such as the first-, second-, and third-order color moments. Textural characteristic parameters were calculated, such as contrast, cross-correlation, energy, and inverse differential moment. The sixth step was fruit yield prediction and analysis. With the fruit tree morphological characteristic parameters, color characteristic parameters, and textural characteristic parameters as inputs, the back-propagation artificial neural network (BPANN) prediction model performed fruit yield prediction, and the UAV measurement accuracy of the morphology and yield information of the fruit trees was evaluated and analyzed.

### 2.2. UAV Measurement Tests for Gathering Orchard Canopy Information

#### 2.2.1. Measurement of Multimodal Information about the Orchard Canopy

In this study, the apple orchard test site for canopy multimodal information measurement was in Yaojiazhuang, Guandao Town, Shandong Province, China (E 120.6352°, N 37.1716°). The measurement test time was from September 15, 2019 to September 25, 2019, when apple trees are at the mature stage. The variety in the apple orchard was Yanfu 3, and the tree age in the measured area was 8–9 years. The measured area was 140 m × 25 m (length × width), including five rows of fruit trees; the row spacing of the fruit trees was 5 m, and; the plant spacing was 4 m. There were few dead trees or young plants in the measured area of this orchard. Because there was plenty of sunlight between 10:00 a.m. and 2:00 p.m. on a sunny day, the impact of tree shadow on image processing was relatively small, so the UAV flight time was between 10:00 and 14:00. The parameters of the Parrot Bluegrass flights are shown in Table 1. The respective flight heights were specified as 22, 32, 43, 53, and 64 m, and the ground sampling distances (GSDs) were 2.13, 3.31, 4.39, 5.43, and 6.69 cm/px. The flight speed was specified as Normal, with the averages of 2.6, 3.7, 4.9, 6.1, and 7.3 m/s. The expected flying time was 5 min 52 s, 2 min 50 s, 1 min 46 s, 1 min 32 s, and 1 min 20 s, respectively. The image front overlap was specified as 80%, and the image side overlap was 80%. The camera was a Parrot Sequoia, which contained a 16-megapixel RGB camera and four 1.2-megapixel spectral channel single-band cameras with a global shutter. The ground control adopted a Parrot Skycontroller 2 controller and Pix4Dcapture flight control software. During the study period, the sky was clear with very few clouds and a wind speed of less than 8 m/s, which met the requirements for remote sensing by UAV.

#### 2.2.2. Preprocessing of Multimodal Information of Orchard Canopy

In this study, Pix4Dmapper software was used to generate 3D point-cloud models of the orchard and vegetation index orthographic images with GRE, RED, REG, and NIR orchard canopy images collected in the measured area. The main process included reading UAV measurement images, specifying coordinate systems, initial processing, and generation of point-clouds, textures, orthophotos, and indices. Pix4Dmapper parameter settings are shown in Table 2. Based on the structure-from-motion (SfM) principle [29], Pix4Dmapper carries out computing keypoints, computing matches, calibration, matching, and point-cloud densification on images, and it finally outputs the PLY file of the orchard point-cloud model, orthographic images, and various JPG files of vegetation index images.

**Figure 1:**
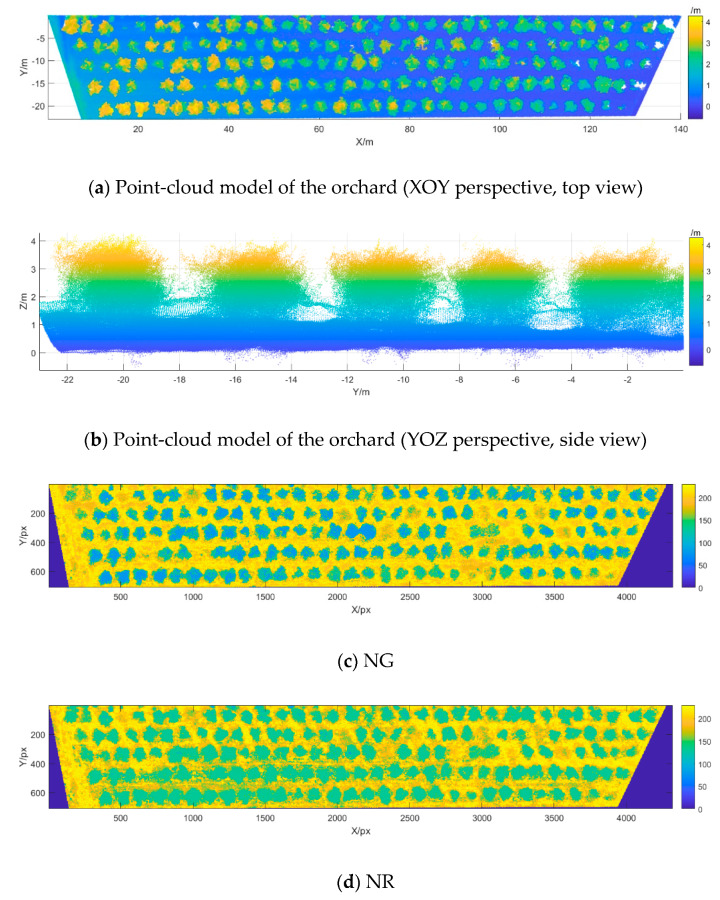

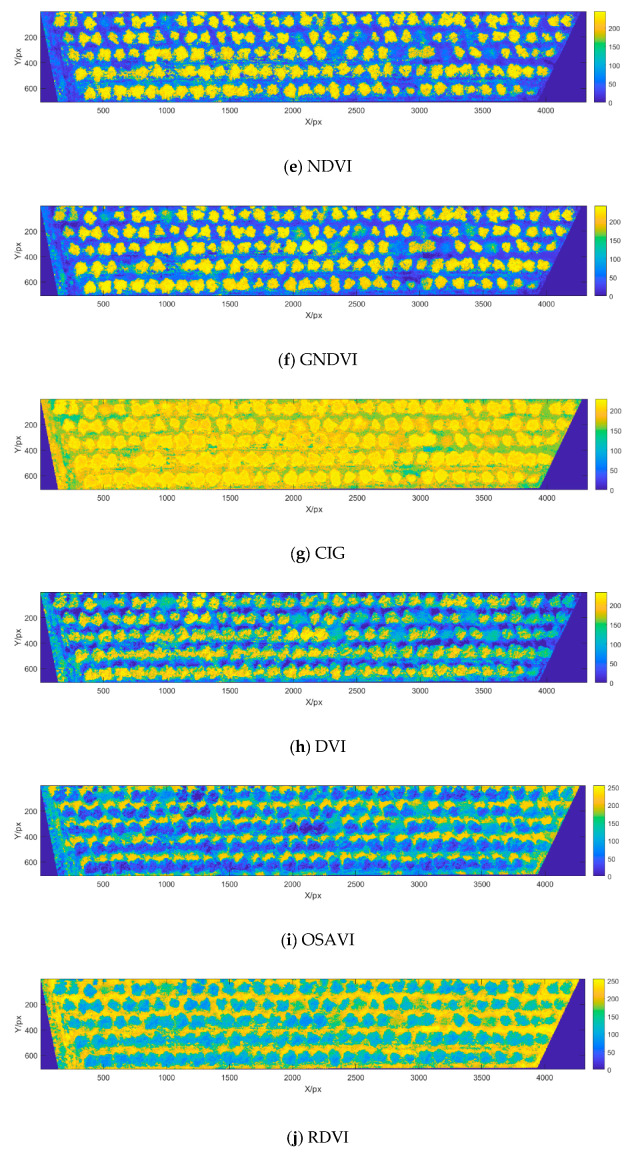

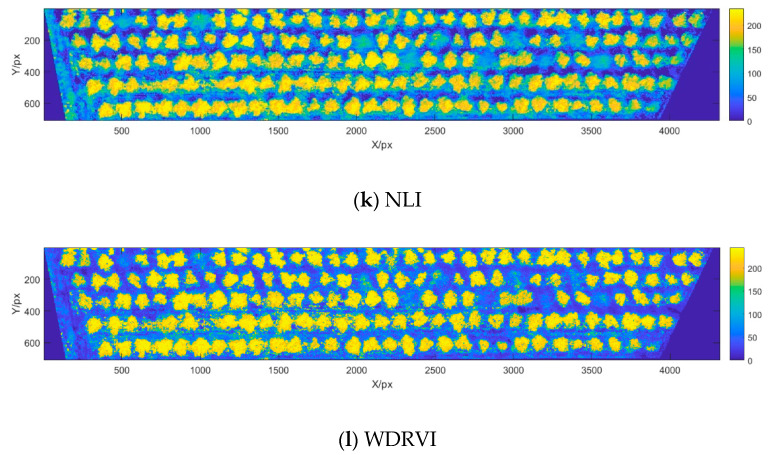
Three-dimensional point-cloud models and vegetation index images of the apple orchard.

Figure 1a shows an image of the 3D point-cloud model from the top view (XOY perspective) of the measured area of the apple orchard. Figure 1b shows the 3D point-cloud model from the side view (YOZ perspective) of the measured area in the apple orchard. Figure 1c–l, respectively, show the NG, normalized red (NR), NDVI, green NDVI (GNDVI), CIG, DVI, optimized soil-adjusted VI (OSAVI), RDVI, nonlinear VI (NLI), and wide-dynamic-range VI (WDRVI) images of the measured area in the apple orchard (grayscale of vegetation index images, convert the index value to 0–255). The formulas to calculate the vegetation indices are shown in Table 3.

### 2.3. Row and Column Detection Methods of the Orchard Canopy

In this study, the row and column detection method of the orchard was proposed: the row and column detection method based on grayscale projection in orchard index images (RCGP). The PLY data of a 3D point-cloud model of the orchard consist of several 3D coordinates, while the JPG data of a vegetation index is a 2D image. The data formats of the two are thus inconsistent. To achieve unified row and column segmentation across the two types of data, it is necessary to perform a conversion of segmentation positions. In this study, the segmentation performance of the RCGP was analyzed. The RCGP based on grayscale projection in orchard index images was selected, and through the row and column segmentation position conversion, the unified segmentation of the orchard multimodal data was achieved.

In the first step, a vegetation index image was grayscale processed, and the cumulative grayscale value for each row was calculated using Equation (1), while the cumulative grayscale value for each column was calculated using Equation (2). In the second step, the cumulative grayscale values for the rows were searched to find the local peaks by probability density estimation, as shown in Equation (3), based on the principle of kernel density estimation, which were taken as the row segmentation positions and were saved to the array *L_2D_*. In the third step, the cumulative grayscale values for the columns were searched to find the local peaks, which were taken as the column segmentation positions and were saved to the cell array *C_2D_*{*i*}:

(1) $$G_{L}(i)=\sum_{j=1}^{n}I(i,j),$$

(2) $$G_{C}(j)=\sum_{i=1}^{m}I(i,j),$$

(3) $${\hat{f}}_{h}(G_{L})=\frac{1}{nh}\sum_{i=1}^{n}K(\frac{G_{L}-G_{L}(i)}{h}),$$

where *I* is the grayscale image of the vegetation index; (*i*, *j*) are the image coordinates; *m* is the number of image rows; *n* is the number of image columns; *G_L_*(*i*) is the cumulative grayscale value of the *i*^th^ row; and *G_C_*(*i*) is the cumulative grayscale value of the *j*^th^ column; ${\hat{f}}_{h}(G_{L})$ is the probability density value of *G_L_*; *h* is the bandwidth, and; *K* is the kernel function.

As shown in Equations (4) and (5), the row segmentation position obtained by the grayscale projection method was converted into a 3D point-cloud model, which could adopt the same row and column segmentation position, thereby realizing the unified row and column segmentation positions with the multimodal data of the orchard. Conversely, the row and column segmentation position obtained by kernel density estimation from the 3D point-cloud model was also converted into vegetation index images:

(4) $$L_{3D}(i)=\frac{|L_{2D}(i)|\times m_{3D}}{m_{2D}},$$

(5) $$C_{3D}\left\{i\}(j\right)=\frac{|C_{2D}\{i\}(j)|\times n_{3D}}{n_{2D}},$$

where *L_2D_* is the array of row segmentation positions obtained by the grayscale projection method; *i* is the row segmentation position number; *m*_2D_ is the number of rows in a vegetation index image, with the unit of px; *m*_3D_ is the Y-axis length of the 3D point-cloud model, with the unit of m; *L*_3D_ is the array of row segmentation positions (converted values) of the 3D point-cloud model; *C_2D_*{*i*} (*j*) is the array of column segmentation positions obtained by the grayscale projection method; *j* is the column segmentation position number; *n_2D_* is the number of columns of the vegetation index image, with the unit of px; *n_3D_* is the X-axis length of the 3D point-cloud model, with the unit of m; and *C_3D_*{*i*} (*j*) is the array of column segmentation positions (converted values) of the 3D point-cloud model.

### 2.4. Methods for Characteristic Value Extraction for Multimodal Data of the Orchard Canopy

To measure orchard canopy information, the morphological characteristic values of fruit trees were extracted through the 3D point-cloud model of the orchard, and the canopy color and textural characteristic values were extracted through the vegetation indices of the orchard.

#### 2.4.1. Calculation Method for Morphological Characteristic Values of Fruit Trees

In this study, the morphological characteristic values of fruit trees extracted mainly included height *H*, projected area *S_XOY_*, and volume *V*. The height *H* is the canopy height of an apple tree, and the maximum vertical height of the Z-axis in a point-cloud model is the canopy height of the highest fruit tree, as shown in Equation (6). The canopy shape of fruit trees is complex, so it is difficult to accurately calculate the canopy volume. There exists a large error in the calculation of the volume using the enclosed volume outside the 3D grids. This is mainly because though the orchard point-cloud models generated by the UAV measurement system have dense point-clouds in the XOY perspective, the point-clouds in the YOZ and XOZ perspectives and other lateral perspectives are relatively sparse. There are also other influencing factors, such as noise interference. In this study, based on the shapes of fruit trees in the measured area, the volume of the canopy was approximated using an outer enclosed ellipsoid circumscribing the canopy. As the point-cloud projection from the XOY perspective had the characteristic of rotation invariance, the point-cloud projection boundary of the XOY perspective was used to calculate the surrounding area *S_XOY_*, as shown in Equation (7). Given that the average trunk height of the fruit trees in the measured area was 0.6 m, the volume of the canopy-circumscribing ellipsoid was calculated using Equation (8). The number of canopy point-clouds *P_ij_* of each fruit tree was counted by Equation (9).

(6) $$H=Z_{\max}{-Z}_{\min},$$

(7) $$S_{XOY}=\frac{1}{2}\sum_{i=1}^{N}\left[x_{i}y_{i+1}-y_{i}x_{i+1}\right],$$

(8) $$V=\frac{4}{3}\pi \frac{\left(H-0.6\right)}{2}\frac{S_{XOY}}{\pi },$$

(9) $$P_{ij}=\sum_{L(i)\sim L(i+1)}^{C\left\{i\right\}(j)\sim C\left\{i\right\}(j+1)}Po\mathrm{int}Cloud,$$

where *H* is the canopy height of a fruit tree, with the unit m; *Z*_max_ is the maximum value on the Z-axis of the point-cloud model of any single apple tree, with the unit m; *Z*_min_ is the minimum value on the Z-axis of the point-cloud model of any single apple tree, with the unit m; *S*_XOY_ is the projection area of a single apple tree’s canopy point-cloud model in the XOY plane, with the unit m^2^; *N* is the number of convex hull vertices; *x_i_* is the *x* coordinate of the *i*^th^ vertex; *x_j_* is the *x* coordinate of the *j^th^* vertex; *y_i_* is the *y* coordinate of the *i*^th^ vertex; *y_j_* is the *y* coordinate of the *j^th^* vertex; *x_i+1_* is the *x* coordinate of the (*i +* 1)^th^ vertex; *y_i+1_* is the *y* coordinate of the (*i +* 1)^th^ vertex; V is the equivalent volume of the circumscribed ellipsoid of the canopy, with the unit m^3^; *P_ij_* is the number of point-clouds in the *i*^th^ row and *j*^th^ column of the fruit tree canopy; *C*{*i*} (*j*) is the value of the initial point on the X-axis of the *i*^th^ row and *j*^th^ column; *C*{*i*} (*j* + 1) is the value of the initial point on the X-axis of the *i*^th^ row and (*j +* 1)^th^ column;; *L*(*i*) is the initial point on the Y-axis of the *i*^th^ column; and *L*(*i* + 1) is the initial point on the Y-axis of the (*i +* 1)^th^ column.

#### 2.4.2. Calculation Method for the Color Characteristic Values of Fruit Trees

The extracted color characteristic values of fruit trees mainly included the first-, second-, and third-order color moments [30]. The calculation formulas are Equations (10)−(12). The color moment is a simple and effective way to express color characteristic values. Since the color information is mainly distributed in low-order moments, the first-, second-, and third-order moments are sufficient to express the color distributions in vegetation index images. There is good evidence that color moments can effectively represent the color distribution in images. The advantages of this method are that it does not require color space quantization and the characteristic vector has a low dimension.

(10) $$\mu =\frac{1}{m\times n}\sum_{i=1:m}^{j=1:n}p(i,j),$$

(11) $$\sigma ={\left(\frac{1}{m\times n}\sum_{i=1:m}^{j=1:n}{\left(p(i,j)-\mu \right)}^{2}\right)}^{\frac{1}{2}},$$

(12) $$s={\left(\frac{1}{m\times n}\sum_{i=1:m}^{j=1:n}{\left(p(i,j)-\mu \right)}^{3}\right)}^{\frac{1}{3}},$$

where *μ* is the first-order color moment of the image; σ is the second-order color moment of the image; *S* is the third-order color moment of the image; *p*(*i*, *j*) is the color value of the image pixel at the coordinate (*i*, *j*); m is the number of rows in the image, and; n is the number of columns in the image.

#### 2.4.3. Calculation Method for the Textural Characteristic Values of Fruit Trees

Grayscale symbiosis matrices or gray-level co-occurrence matrices (GLCMs) were used to express the textural characteristic values of vegetation index images. Haralick et al. [31] were the first to convert grayscale values into textural information based on GLCMs. GLCMs have the advantages of simple processing, good discrimination, and strong adaptability. They have become an important method for analyzing image textural characteristic values [31]. The characteristics of GLCMs mainly include contrast (CON), correlation (COR), angular second-order moment (ASM), and entropy; the calculation formulas are Equations (13)−(16). CON reflects the image clarity and texture depth, and its value indicates the local grayscale correlation in an image. COR is a measure of the similarity of spatial GLCM elements in the row or column direction, and its value indicates the local grayscale correlation in an image. ASM reflects the uniformity of grayscale distribution and the thickness of texture in an image. The more concentrated the grayscale distribution in an image or the coarser the texture, the greater the ASM value; conversely, the more discrete the grayscale distribution or the finer the texture, the lower the ASM value. INM reflects the homogeneity of image texture and measures the local change of image texture; the larger the inverse difference moment, the smaller the change in image texture between different regions, and the greater the local uniformity. These variables are calculated as follows:

(13) $$CON=\sum_{i=0:N-1}^{j=0:N-1}{\left|i-j\right|}^{2}p(i,j),$$

(14) $$COR=\sum_{i=0:N-1}^{j=0:N-1}\frac{(i\times j\times p(i,j))-\sum_{i=0:N-1}^{j=0:N-1}i\times p(i,j)\sum_{i=0:N-1}^{j=0:N-1}j\times p(i,j)}{\sum_{i=0:N-1}^{j=0:N-1}p(i,j)(i-\sum_{i=0:N-1}^{j=0:N-1}i\times p(i,j){)}^{2}\times \sum_{i=0:N-1}^{j=0:N-1}p(i,j)(j-\sum_{i=0:N-1}^{j=0:N-1}j\times p(i,j){)}^{2}},$$

(15) $$ASM=\sum_{i=0:N-1}^{j=0:N-1}p{\left(i,j\right)}^{2},$$

(16) $$INM=\sum_{i=0:N-1}^{j=0:N-1}\frac{p(i,j)}{1+\left|i-j\right|},$$

where *CON* is the image contrast; *COR* is the image correlation; *ASM* is the angular second moment of the image; *INM* is the inverse difference moment; (*i, j*) are two different grayscale values; *p*(*i*, *j*) is the normalized probability of the occurrence of pixel pair (*i, j*); and *N* is the grayscale level of the image.

### 2.5. Measurement Models and Data Analysis Methods for Orchard Canopy Information

#### 2.5.1. Results and Analysis Methods for Row and Column Detection in the Orchard Canopy

Next, the segmentation accuracy (correction rate) and false/erroneous recognition rate of the row and column detection method based on RCGP were statistically analyzed; the calculation formulas are Equations (17) and (18). Specifically, the segmentation performance based on the NG vegetation index images was calculated when GSD was 2.13, 3.31, 4.39, 5.43, and 6.69 cm/px.

(17) $$C=\frac{NC}{N}\times 100\%,$$

(18) $$E=\frac{NE}{N}\times 100\%,$$

where *C* is the correction/accuracy rate, reported as %; *NC* is the number of correctly segmented regions; *N* is the number of total regions; *E* is the erroneous recognition (false recognition) rate, reported as %; and *NE* is the number of erroneously recognized regions (including repeat recognition regions and erroneously recognized regions).

#### 2.5.2. Results and Analysis Methods for the Measurement of Orchard Morphological Characteristic Values

First, a tape measure was used to manually measure the total height of the canopy, trunk height, canopy width, and fruit yield of the apple tree in the measurement area. Each measurement was done three times, and the average value was taken. Based on the average width of a canopy, the area of canopy projection was calculated. According to Equations (6)–(8), the volume of the circumscribing ellipsoid of the apple tree canopy was calculated as the measured circumscribing volume of the apple tree canopy.

Through the methods in Section 2.4.1, the 3D point-cloud model of the orchard was extracted. The height of fruit tree *H*, the projected area *S_XOY_*, and the volume *V* were extracted. The correctness (accuracy) of fruit tree morphological characteristic values was calculated when GSD was 2.13, 3.31, 4.39, 5.43, and 6.69 cm/px. The coefficient of determination (R^2^), root mean square error (RMSE), and relative average deviation (RAD) were used to analyze the measurement errors of fruit tree morphological characteristic values.

#### 2.5.3. Results and Analysis Methods for Orchard Yield Measurement

In this study, BPANN was used for modeling. Specifically, the independent variables in the model included three morphological characteristic values, three color characteristic values, and four textural characteristic values. NG, NR, NDVI, GNDVI, DVI, CIG, OSAVI, RDVI, WDRVI, and NLI were used for extracting color characteristic values and textural characteristic values. The actual measured value of fruit yield was taken as the dependent variable. Statistical analysis was performed on the model accuracy of fruit yield measurement when GSD was 2.13, 3.31, 4.39, 5.43, and 6.69 cm/px. To evaluate the accuracy of the model and test the performance of the model in fruit yield prediction, the R^2^, RMSE, and RAD were used.

## 3. Results and Discussion

### 3.1. Characteristic Extraction from Multimodal Data of the Orchard Canopy

**Figure 2:**
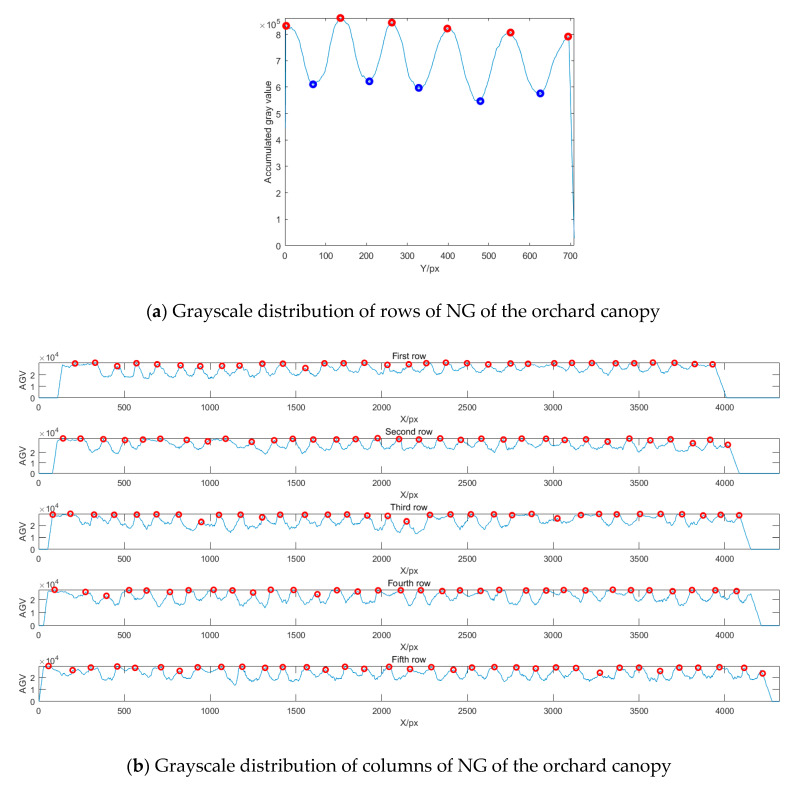
Row and column detection with multimodal data of orchard canopy.

The grayscale image of the NG index was used for row and column detection in the orchard. Figure 2a shows the Y-axis grayscale projection of the orchard vegetation index image, which is the cumulative grayscale value for all the rows in the grayscale image of the index NG. The peak and valley values of the Y-axis cumulative grayscale values are marked with red and blue circles, respectively, and the local peak values of the Y-axis cumulative grayscale values were taken as the row segmentation positions. The row segmentation positions were saved into the array *L*; the images of each row’s area were also saved. Likewise, the X-axis in the image for each row’s area was in turn grayscale projected, and the local peak values in the cumulative grayscale values and their corresponding X-values were searched. As shown in Figure 2b, the local peak values of the X-axis cumulative grayscale values for each row are marked with red circles, and the local grayscale peak values of the X-axis were taken as the column segmentation positions, which were saved into the cell array *C*{*i*}.

**Figure 3:**
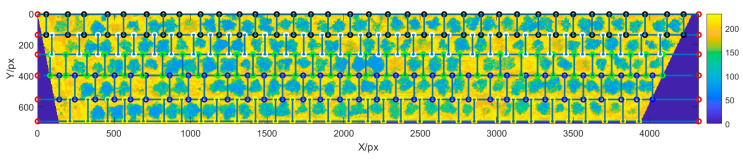
Row and column detection results with orchard canopy data.

The measured area in this study had a total of five rows, and there were, respectively, 30, 31, 32, 33, and 34 apple trees in rows 1 to row 5. This gave a total of 160 apple trees, including eight dead trees that had only a trunk or had few leaves. Figure 3 shows the row and column detection results based on grayscale projection in orchard index images. Based on the RCGP method, the row segmentation positions were accurately detected (accuracy rate 100%) under various GSDs.

In this study, the correction/accuracy rate *C* and the erroneous-recognition rate *E* were calculated with Equations (17) and (18). Statistical analysis of the RCGP method was performed, and the performance of column detection when *GSD* was 2.13, 3.31, 4.39, 5.43, and 6.69 cm/px was compared.

**Figure 4:**
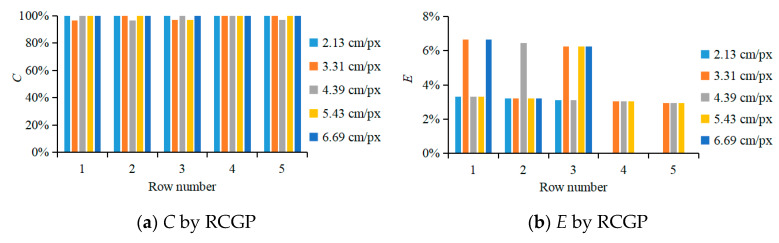
Performance of column detection with orchard canopy data.

In Figure 4, when using the RCGP detection method and GSD values of 2.13, 3.31, 4.39, 5.43, and 6.69 cm/px, the average correction/accuracy rates of column detection in the grayscale image of NG index were 100.00%, 98.71%, 98.77%, 99.38%, and 100.00%, respectively, and the average erroneous-recognition rates were 1.94%, 4.42%, 3.78%, 3.76%, and 3.23%. With the RCGP detection method, the accuracy rate and misrecognition rate in column detection were not significantly affected by GSD. 

According to the column detection statistics, the main reason for false recognition by RCGP lay in the misidentification of the border area. Because the orchard point-cloud model generated by the UAV was matched according to the key points of the images collected from different waypoints, there were very few leaves on the dead trees, resulting in very few point-clouds generated in these locations/areas, which in turn affected the performance of the 3D detection method. On the other hand, the RCGP detection method detected the dead trees with few leaves, so the column detection performance using this method was optimal, accurate, and stable.

### 3.2. Calculation and Error Analysis of Orchard Canopy Morphological Information

With the RCGP row and column detection method, the row and column segmentation positions in vegetation index images were determined. The row and column segmentation positions in the 3D point-cloud model were obtained using Equations (4) and (5). The unified segmentation positions in the fruit tree region were used. The morphological parameters of the fruit tree canopy were calculated using Equations (6)–(9), the color characteristic values of the vegetation indices of the fruit tree canopy were calculated using Equations (10)–(12), and the textural characteristic values of the vegetation indices of the fruit tree canopy were calculated using Equations (13)–(16).

**Figure 5:**
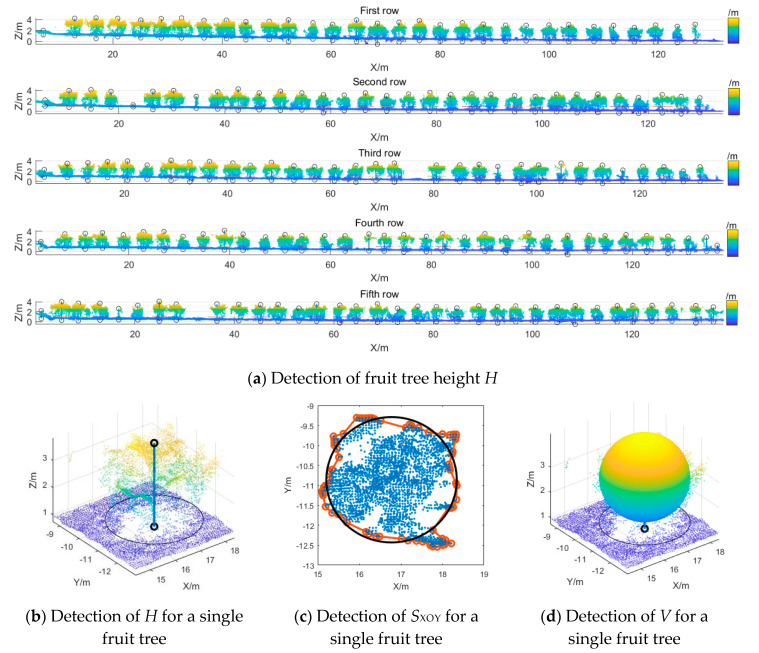
Detection of morphological characteristic values of orchard canopy.

Figure 5a shows the detection results for the fruit tree canopy height *H*, where the highest point and lowest point in each fruit tree area were detected, and the distance between the two points was the height of the fruit tree, as shown in Figure 5b. Figure 5c shows the detection results for *S*_XOY_, the projected area of the fruit tree canopy, which is the area enclosed by the projected boundary of the point-cloud model in the XOY perspective, the red circle and the column mark the boundary convex hull point set. Taking the coordinates with average values of the X-axis and Y-axis of the point-cloud model as the center point, the equivalent circle of canopy projection was drawn (the black circle in Figure 5c). The area of this circle is equal to *S*_XOY_. In this study, the average value of the trunk of the fruit trees was 0.6 m, and the shape of the canopy was similar to that of an ellipsoid. Hence, based on the tree height *H*, the height of the trunk, and the projected canopy area *S*_XOY_, the volume of the canopy-circumscribing ellipsoid of fruit tree was calculated, as shown in Figure 5d.

Statistical analysis was performed on the measurement results of morphological parameters of the fruit tree canopy, including *H*, *S_XOY_*, and *V*, as shown in Table 4. When GSD was 2.13, 3.31, 4.39, 5.43, and 6.69 cm/px, upon comparing the hand-measured *H* values of the canopy and the values measured with the UAV, the *R*^2^ value was, respectively 0.94, 0.92, 0.91, 0.88, and 0.85; the RMSE was 0.08 m, 0.10 m, 0.09 m, 0.10 m, and 0.14 m; and the RAD_avg_ was 1.72%, 1.77%, 1.78%, 2.67%, and 3.42%. Upon comparing the hand-measured values of *S*_XOY_ of the fruit tree canopy and the values measured with the UAV, the R^2^ value was, respectively, 0.94, 0.91, 0.86, 0.86, and 0.79; the RMSE was 0.72 m^2^, 0.94 m^2^, 1.10 m^2^, 1.13 m^2^, and 1.39 m^2^; and the RAD_avg_ was 4.33%, 5.98%, 7.19%, 7.75%, and 9.87%. Upon comparing the hand-measured values of *V* of the fruit tree canopy and the values measured with the UAV, R^2^ was 0.91, 0.86, 0.85, 0.83, and 0.80, respectively; *RMSE* was 1.41 m^3^, 1.63 m^3^, 1.64 m^3^, 1.91 m^3^, and 2.21 m^3^; and RAD_avg_ was 7.90%, 8.28%, 11.90%, 12.61%, and 13.69%. Thus, the statistical results showed that GSD had a significant impact on the accuracy and correlation of the calculated 3D morphological parameters in the point-cloud model of the orchard: as GSD increased, R^2^ decreased and RMSE increased. When GSD was 2.13, 3.31, 4.39, 5.43, and 6.69 cm/px, the point-cloud density in the 3D point-cloud model was, respectively, 364.77 points/m^3^, 75.55 points/m^3^, 32.01 points/m^3^, 17.22 points/m^3^, and 8.65 points/m^3^. As GSD directly affected the density of the orchard point-cloud model, the average point-cloud density had a significant decreasing trend as GSD increased, resulting in an increasing error in the measurement of canopy morphological parameters. GSD had a greater impact on the measured values of *S*_XOY_ and *V*, and a small amount of noise caused a large error in measurements. Specifically, the RAD_max_ range of *S*_XOY_ was 19.48–35.78% and the RAD_max_ range of *V* was 37.36–55.99%.

The *S*_XOY_ of the fruit tree canopy had a higher measurement error than *H*, which was mainly because the *S*_XOY_ values were calculated from the actual measured widths of fruit trees. Although the width of the fruit tree was the average of multiple measurements, the width of a fruit tree could easily be measured wrong by hand, leading to a certain difference from the calculated value. In the UAV measurement method, as long as the fruit tree area is accurately segmented, the canopy boundary point set can be quickly found through the boundary point spacing search method. Although *S*_XOY_ can be calculated from the boundary convex hull point set, it requires an accurate point-cloud model. The fruit tree canopy *V* had a higher measurement error than *H* and *S*_XOY_, which was mainly due to the use of canopy-circumscribing ellipsoids to approximate the canopy volume of fruit trees. There were errors in both UAV measurements and manual measurements of *H* and *S*_XOY_, which led to a relatively large error in *V*.

### 3.3. Orchard Yield Prediction and Error Analysis

Three color moment characteristic values and four textural characteristic values of the fruit tree canopy vegetation indices and four morphological characteristic values of the 3D point-cloud model were used as the input characteristic variables in the orchard yield prediction model. The actual measured yield values of fruit trees were taken as the model output variables for constructing the fruit yield prediction model. The canopy vegetation indices of fruit trees were NG, NR, NDVI, GNDVI, DVI, CIG, OSAVI, RDVI, WDRVI, and NLI (Table 3). Six input modes were used for the fruit yield prediction model, named Inputs 1–6. Specifically, Input 1 included 10 input characteristics, i.e., color moment characteristic values and textural characteristic values of one vegetation index and the 3D morphological characteristic values. Input 2 included four input characteristics: the 3D morphological characteristic values of the fruit trees. Input 3 included 30 input characteristics, i.e., color moment characteristic values of all 10 vegetation indices. Input 4 included 40 input characteristics, i.e., textural moment characteristic values of 10 vegetation indices. Input 5 included 70 input characteristics, i.e., color moment characteristic values and textural moment characteristic values of all 10 vegetation indices. Input 6 included 110 input characteristics, i.e., color moment characteristic values and textural moment characteristic values of all 10 vegetation indices and the morphological characteristic values of the 3D point-cloud model.

BPANN was used to establish the fruit yield prediction model. A three-layer network structure was used, where the excitation function from the input layer to the hidden layer was a tansig function and that from the hidden layer to the output layer was a purelin function. The learning function was a function of gradient descent with momentum weight, and the training function was the Levenberg–Marquardt algorithm. The empirical formula h = sqrt(m + n) + a was used to calculate and select the node number of the hidden layer, where *h* is the number of hidden layers in the BPANN, *m* is the number of input layers, *n* is the number of output layers, and *a* is the adjustment constant (a = 1–10). The percentages of training, validation, and testing data of BPANN model is 60%, 15%, and 25%, respectively. The performance of the fruit yield prediction model under different GSDs was statistically analyzed.

**Figure 6:**
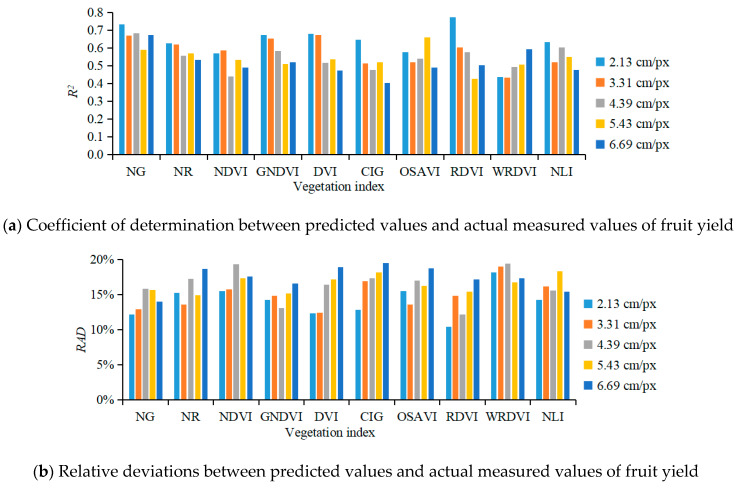
Performance parameters of the fruit yield prediction model using various vegetation indices.

When GSD was 2.13, 3.31, 4.39, 5.43, and 6.69 cm/px, for Input 1, in which the characteristic values of vegetation indices NG, NR, NDVI, GNDVI, DVI, CIG, OSAVI, RDVI, WDRVI, and NLI were separately used in the BPANN prediction model, R^2^ ranged from 0.59–0.73, 0.54–0.63, 0.44–0.59, 0.51–0.67, 0.48–0.68, 0.40–0.65, 0.49–0.66, 0.43–0.77, 0.44–0.59, and 0.48–0.63, respectively (Figure 6a); RAD ranged from 12.18–15.86%, 13.59–18.69%, 15.49–19.33%, 13.12–16.59%, 12.32–18.95%, 12.82–19.48%, 13.60–18.74%, 10.47–17.15%, 16.76–19.45%, and 14.26–18.31% (Figure 6b); RMSE ranged from 9.94–12.23 kg, 11.65–13.10 kg, 12.17–14.68 kg, 10.91–13.90 kg, 10.79–14.21 kg, 11.37–14.74 kg, 12.90–15.45 kg, 9.18–14.10 kg, 12.76–16.85 kg, and 11.53–14.88 kg. The statistical data showed that when the color moment characteristic values, textural characteristic values, and 3D morphological characteristic values of one vegetation index were input into the yield prediction model, the correlation between the predicted yield value and the measured value was not significant, and the differences in the prediction performance between various vegetation indices were not significant. The main reason was that one vegetation index has limited information, and the correlation between fruit yield and fruit tree morphology is not significant enough, which led to the inadequate performance of the fruit yield prediction model.

**Figure 7:**
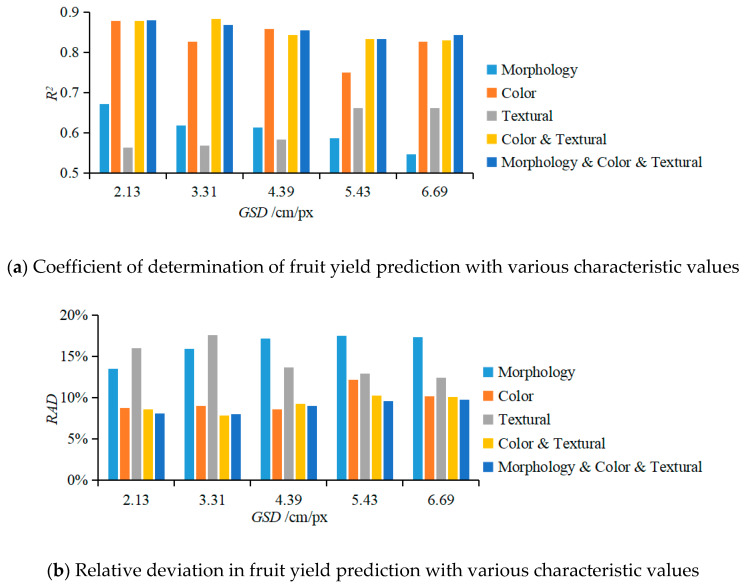
Performance parameters of the fruit yield prediction model with various characteristic values.

When GSD was 2.13, 3.31, 4.39, 5.43, and 6.69 cm/px, for Input 2, the R^2^ of the BPANN prediction model was, respectively, 0.67, 0.62, 0.61, 0.59, and 0.55 (Figure 7a); RAD was 13.51%, 15.97%, 17.21%, 17.47%, and 17.31% (Figure 7b); and RMSE was 10.92 kg, 11.84 kg, 12.12 kg, 12.78 kg, and 12.85 kg. Statistical analysis showed that when the fruit tree morphological characteristic values were used to predict the fruit yield, the model correlation was not significant, and as GSD increased, the performance of this prediction model worsened, with an increase in the RAD. This was mainly because GSD affected the accuracy of the orchard 3D point-cloud model, which led to an increase in the calculation error of the morphological characteristic values; additionally, the morphological characteristic values are not the only determinants of fruit yield.

When the GSD was 2.13, 3.31, 4.39, 5.43, and 6.69 cm/px, for Input 3, the *R*^2^ of the BPANN prediction model was, respectively, 0.88, 0.83, 0.86, 0.75 and 0.83, as shown in Figure 7a; the *RAD* values were respectively 8.74%, 9.01%, 8.56%, 12.15% and 10.17%, as shown in Figure 7b; and the *RMSE* values were respectively 7.71 kg, 7.94 kg, 7.20 kg, 9.69 kg and 8.29 kg. The statistical data showed that when the color characteristic values of fruit tree vegetation indices were used to predict the fruit yield, the model correlation was significant; only when the *GSD* was 5.43 cm/px, the significance was relatively low. Under different *GSD*s, the performance of the prediction model was similar. The performance of the prediction model outperformed that of Input 1, where single index characteristic values were used for model prediction. The color characteristic values of the fruit tree canopy vegetation index can be used to predict the yield of fruit trees.

When GSD was 2.13, 3.31, 4.39, 5.43, and 6.69 cm/px, for Input 4, the R^2^ of the BPANN prediction model was, respectively, 0.56, 0.57, 0.58, 0.66, and 0.66 (Figure 7a); RAD was 16.03%, 17.59%, 13.66%, 12.91%, and 12.47% (Figure 7b); and RMSE was 13.13 kg, 12.78 kg, 12.71 kg, 11.25 kg, and 12.10 kg. The statistical analysis showed that when the textural characteristic values of fruit tree vegetation indices were input to predict the fruit yield, the model correlation was not significant. As GSD increased, the prediction model performance improved. This was mainly because GSD affects the textures of orchard vegetation index images, so as GSD increased, the canopy area in the vegetation index image became more blurred, while the edges became clearer. However, using the textural characteristic values of vegetation indices to predict the yield of fruit trees has a relatively large error, so textural characteristic values cannot be applied to yield prediction of fruit trees.

When GSD was 2.13, 3.31, 4.39, 5.43, and 6.69 cm/px, for Input 5, the R^2^ of the BPANN prediction model was, respectively, 0.88, 0.88, 0.84, 0.83, and 0.83 (Figure 7a); RAD was 8.57%, 7.85%, 9.23%, 10.23%, and 10.10% (Figure 7b); and RMSE was 8.35 kg, 6.74 kg, 8.18 kg, 8.62 kg, and 8.28 kg. The statistical analysis showed that when the color characteristic values and textural characteristic values of fruit tree vegetation indices were input to predict the fruit yield, the model correlation was significant; as GSD increased, the performance of this prediction model worsened. The model performance was similar to that of Input 3. Hence, the color characteristic values and textural characteristic values of canopy vegetation indices can be used to predict fruit yield, but considering the prediction performance, textural characteristic values can be ignored, and the color characteristic values of canopy vegetation indices can be used by themselves to predict fruit yield.

When GSD was 2.13, 3.31, 4.39, 5.43, and 6.69 cm/px, for Input 6, the R^2^ of the BPANN prediction model was, respectively, 0.88, 0.87, 0.86, 0.83, and 0.84 (Figure 7a); RAD was 8.10%, 8.05%, 9.03%, 9.56%, and 9.76% (Figure 7b); and RMSE was 6.90 kg, 7.03 kg, 7.25 kg, 7.96 kg, and 7.93 kg. The statistical analysis showed that when the color characteristic values and textural characteristic values of fruit tree vegetation indices and the morphological characteristic values of the point-cloud model were input to predict fruit yield, the model correlation was significant, and the prediction model performance was similar to that of Input 3 and Input 5. Therefore, the input characteristics of Input 6 can be applied to fruit yield prediction.

Based on the above analysis, using the color characteristic values of canopy vegetation indices of fruit trees to predict the yield of fruit trees performed better than using the textural and morphological characteristic values. This was mainly because given the UAV measurement mode used in this study, the generated orthophoto images of orchard canopy had a large amount of color characteristic information, while the textural characteristic values of the canopy were relatively blurred, so the amount of information they bore was relatively small. On the other hand, the morphological characteristic values were affected by the accuracy of the 3D point-cloud model, and the morphological characteristic values could not adequately predict the fruit yield. Thus, using color characteristic values of multiple vegetation indices to predict yield performance is superior to using that of one vegetation index, which is mainly due to the screening of vegetation indices in this study, in which vegetation indices with more obvious characteristic advantages were selected. Multiple vegetation indices contained more canopy information and thus had better prediction performance. This method can be applied to yield prediction of any modern standardized apple orchards.

## 4. Conclusions

This study proposes a UAV multimodal information-based method for the measurement of canopy morphological information and for yield prediction of apple orchards. The main conclusions are as follows:

1. In this study, a row and column detection method based on grayscale projection in orchard index images, RCGP, is proposed. It allows row and column segmentation using multimodal information of fruit tree canopies in modern standardized apple orchards. The results showed that using the RCGP method, the correction/accuracy rate of row detection in the orchard was 100.00%. Using the RCGP method, when GSD was 2.13, 3.31, 4.39, 5.43, and 6.69 cm/px, the average correction/accuracy rates of column detection based on the grayscale images of NG index were, respectively, 100.00%, 98.71%, 98.77%, 99.38%, and 100.00%, and the average misrecognition rates were 1.94%, 4.42%, 3.78%, 3.76%, and 3.23%. The RCGP detection method can detect dead trees with few leaves, so the column detection performance using this method was accurate and stable.
2. A method for measuring canopy morphological information of fruit trees based on the 3D point-cloud model of orchards is established. The results show that when GSD was 2.13, 3.31, 4.39, 5.43, and 6.69 cm/px, comparing the hand-measured values of fruit tree canopy height *H* and the UAV-measured values yielded an R^2^ of 0.85–0.94, a RMSE of 0.08–0.14 m, and a RAD_avg_ of 1.72–3.42%; comparing the hand-measured values of fruit tree canopy *S*_XOY_ and the UAV-measured values yielded an R^2^ of 0.79–0.94, a RMSE of 0.72–1.39 m^2^, and a RAD_avg_ of 4.33–9.87%; comparing the hand-measured values of fruit tree canopy *V* and the UAV-measured values yielded an R^2^ of 0.80–0.91, a RMSE of 1.41–2.21 m^3^, and a RAD_avg_ of 7.90–13.69%.
3. The BPANN prediction models for measuring orchard yield are established, when the color moment characteristic values and textural moment characteristic values of all 10 vegetation indices and the morphological characteristic values of the 3D point-cloud model were input to predict fruit yield, the results show that when GSD was 2.13, 3.31, 4.39, 5.43, and 6.69 cm/px, the R^2^ of the BPANN prediction model was, respectively, 0.88, 0.87, 0.86, 0.83, and 0.84, RAD was 8.10%, 8.05%, 9.03%, 9.56%, and 9.76% and RMSE was 6.90 kg, 7.03 kg, 7.25 kg, 7.96 kg, and 7.93 kg. The model correlation was significant, which can be applied to fruit yield prediction.

The row and column detection method proposed in this study is suitable for segmentation using multimodal canopy data in modern standardized orchards, which lays a good foundation for orchard canopy information measurement. The information of canopy morphological and vegetation index characteristic values could be extracted at the same time by this method, which provided more effective information for fruit yield prediction and improves the accuracy of fruit yield prediction. This study established a canopy morphological measurement method and a yield prediction method for fruit trees, which can provide a theoretical basis and technical reference for canopy information measurements in modern standardized orchards and will be valuable in the production management of modern standardized orchards.

## Figures and Tables

**Table 1:** Flight, camera, and ground control parameters.

Pix4Dcapture	Values
Flight altitude	22 m, 32 m, 43 m, 53 m, 64 m
Flight speed	Normal: 22 m (2.6 m/s), 32 m (3.7 m/s), 43 m (4.9 m/s), 53 m (6.1 m/s), 64 m (7.3 m/s)
Image overlap	Front overlap: 80%; side overlap: 80%
Camera	Parrot Sequoia, 16 million pixels, image format: JPG
Ground control	Pix4Dcapture: flight planning, Parrot Skycontroller 2
Weather	Clear, little wind

**Table 2:** Summary of parameters used for SfM processing in Pix4Dmapper.

Pix4Dmapper	Parameters
Coordinate Systems	Image coordinate system: WGS 84 (EGM 96 Geoid); unit: m Output coordinate system: WGS 84/UTM zone 51N (EGM 96 Geoid)
Initial Processing	Keypoint image scale: Full Calibration Method: Standard Internal Parameters Optimization: All External Parameters Optimization: All Rematch: Auto, yes
Point cloud densification	Image scale: 1/2 of original image size Point density: Optimal Minimum number of matches: 3 Export format: PLY
DSM, orthographic image, and indices	Indices: NG, NR, NDVI, GNDVI, DVI, CIG, OSAVI, RDVI, WDRVI, NLI

**Table 3:** Vegetation indices and their calculation formulas.

Vegetation Index	Formula	Vegetation Index	Formula
NG	GRE/(NIR + RED + GRE)	NR	RED/(NIR + RED + GRE)
NDVI	(NIR − RED)/(NIR + RED)	GNDVI	(NIR − GRE)/(NIR + GRE)
DVI	NIR − RED	CIG	NIR/GRE − 1
OSAVI	1.16 (NIR − RED)/(NIR + RED + 0.16)	RDVI	(NIR − RED)/(NIR + RED) ^1/2^
NLI	(NIR^2^ − RED)/(NIR^2^ + RED)	WDRVI	(0.2NIR − RED)/(0.2NIR + RED)

**Table 4:** Analysis of the measurement results of morphological characteristic parameters of the orchard canopy.

Morphological Parameter	GSD	R2	RMSE	RADmax	RADmin	RADavg
*H*	2.13 cm/px	0.94	0.08 m	8.04%	0.03%	1.72%
	3.31 cm/px	0.92	0.10 m	9.74%	0.00%	1.77%
	4.39 cm/px	0.91	0.09 m	9.51%	0.00%	1.78%
	5.43 cm/px	0.88	0.10 m	8.04%	0.04%	2.67%
	6.69 cm/px	0.85	0.14 m	10.93%	0.01%	3.42%
*S_XOY_*	2.13 cm/px	0.94	0.72 m^2^	19.48%	0.09%	4.33%
	3.31 cm/px	0.91	0.94 m^2^	24.72%	0.00%	5.98%
	4.39 cm/px	0.86	1.10 m^2^	38.87%	0.03%	7.19%
	5.43 cm/px	0.86	1.13 m^2^	40.54%	0.02%	7.75%
	6.69 cm/px	0.79	1.39 m^2^	35.78%	0.05%	9.87%
*V*	2.13 cm/px	0.91	1.41 m^3^	37.36%	0.36%	7.90%
	3.31 cm/px	0.86	1.63 m^3^	35.91%	0.38%	8.28%
	4.39 cm/px	0.85	1.64 m^3^	35.78%	0.14%	11.90%
	5.43 cm/px	0.83	1.91 m^3^	49.79%	0.16%	12.61%
	6.69 cm/px	0.80	2.21 m^3^	55.99%	0.35%	13.69%
